# Prevention the formation of biofilm on orthopedic implants by melittin thin layer on chitosan/bioactive glass/vancomycin coatings

**DOI:** 10.1007/s10856-021-06551-5

**Published:** 2021-06-22

**Authors:** Vahid Zarghami, Mohammad Ghorbani, Kamran Pooshang Bagheri, Mohammad Ali Shokrgozar

**Affiliations:** 1 Institute for Nanoscience & Nanotechnology, Sharif University of Technology, Tehran, Iran; 2 Department of Materials Science and Engineering, Sharif University of Technology, Tehran, Iran; 3 Venom & Biotherapeutics Molecules Laboratory, Biotechnology Department, Biotechnology Research Center, Pasteur Institute of Iran, Tehran, Iran; 4 National Cell Bank of Iran, Pasteur Institute of Iran, Tehran, Iran

## Abstract

Methicillin-resistant and Vancomycin-resistant *Staphylococcus aureus* bacteria (MRSA and VRSA, respectively) can seriously jeopardizes bone implants. This research aimed to examine the potential synergistic effects of Melittin and vancomycin in preventing MRSA and VRSA associated bone implant infections. Chitosan/bioactive glass nanoparticles/vancomycin composites were coated on hydrothermally etched titanium substrates by casting method. The composite coatings were coated by Melittin through drop casting technique. Melittin raised the proliferation of MC3T3 cells, making it an appropriate option as osteoinductive and antibacterial substance in coatings of orthopedic implants. Composite coatings having combined vancomycin and Melittin eliminated both planktonic and adherent MRSA and VRSA bacteria, whereas coatings containing one of them failed to kill the whole VRSA bacteria. Therefore, chitosan/bioactive glass/vancomycin/Melittin coating can be used as a bone implant coating because of its anti-infective properties.

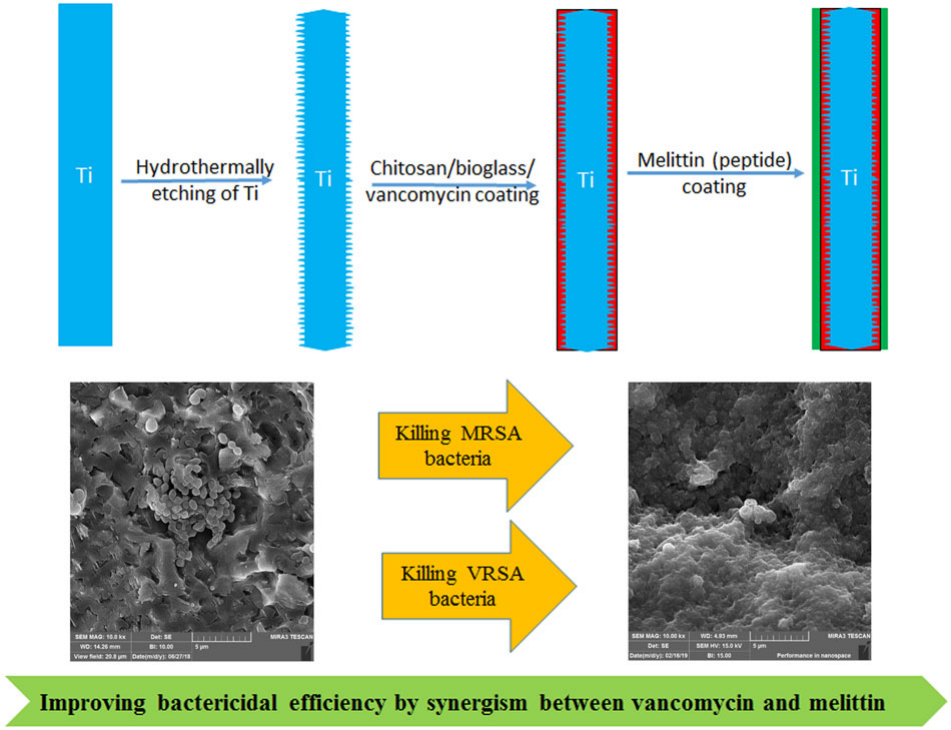

## Introduction

Methicillin-Resistant Staphylococcus Aureus (MRSA) bacterial infection and biofilm formation on the surfaces of prosthesis are the main challenges of orthopedic surgeries [1, 2]. Antimicrobial peptides (AMPs) have been of great interest to dealing with biofilm associated infections and antibiotic resistant bacteria [3, 4]. Melittin, an antimicrobial peptide of 26 amino acid residues, encourages pores in membranes of resistant bacteria cells and hindering biofilm formation [4].

Vancomycin, as a hydrophilic glycopeptide antibiotic, has been broadly applied for eradication of S. aureus bacterial infection and prevention of biofilm development on the implants [5]. Prolonged consumption of customary antibiotics continually brings about bacterial resistance as well [6]. Vancomycin-Resistant Staphylococcus aureus (VRSA) bacteria are today gaining around worldwide in spite of being less prevalent [7].

VRSA infections have been reported from different countries [8–10]. Accordingly, discovery or design new promising antimicrobial agents is vitally necessitated due to the world threat for the outbreak of VRSA isolates [8].

Chitosan/bioactive glass composite coatings have integrated the versatility of chitosan with sturdiness and bioactivity of bioactive glass particles [11]. Accordingly, such composites have appeared in recent years as a novel group of implant coatings which featuring fascinating characteristics enhanced mechanically, physicochemically, and biologically [12–15].

This study hypothesized to improve bactericidal efficacy of Chitosan/bioactive glass/vancomycin antibiotic by applying Melittin peptide thin layer as second layer on it. Therefore, Melittin thin layer was casted on Chitosan/bioactive glass/vancomycin composite coating. Then physiochemical cell proliferation, differentiation, and antibacterial features of coatings and control groups against MRSA and VRSA bacteria were investigated.

## Materials and methods

### Coating process

#### Chitosan-based composite coating

Chitosan powders with 75–85% degree of deacetylation and a low molecular weight (Mw = 50,000–190,000 Da; Sigma-Aldrich, Germany) were dissolved at 6 g/l in 0.25% acetic acid aqueous solution. BGNs and vancomycin powders were poured into the CS solution for preparing composite suspensions with proportionate concentrations of nanoparticles and antibiotic. The solution was subjected to magnetic agitation within 24 h and then sonicated for 30 min for dispersal and homogenization immediately prior to precipitation. The solutions (25 µl) were drop-cast over the biomedical grade titanium foils (Alfa Aesar, USA) with dimensions of 5 × 5 × 0.7 mm, and then dried at enclosed situations for 24 h.

To improve the adhesion of chitosan coatings to substratum, samples were exposed to hydrothermal etching in 0.2 M sulfuric acid aqueous solution at 170 °C for 2 h prior to coating. The etching procedure was done by a Teflon-lined stainless steel autoclave filled up to 70%.

#### Antimicrobial peptide coating

Melittin (Sigma-Aldrich: USA) with 85% purity was dissolved and vortexed in ultra-pure water. Then, Melittin solution was drop-casted over coatings and dried at ambient setting for 3 h.

#### Experimental groups

Table 1 lists the experimental groups and their composition.

**Table 1:** The composition of experimental groups

Row	Group name	Composition			
		Chitosan (µg/µl)	Bio glass (µg/µl)	Vancomycin (µg/µl)	Melittin (µg/µl)
1	Ti	–	–	–	–
2	CS	6	–	–	–
3	CBG	6	6	–	–
4	CBVA	6	6	1.28	–
5	CBME	6	6	–	0.032
6	CBVME	6	6	0.16	0.016

*Ti* Titanium, *CS* Chitosan, *CBG* Chitosan/Bioactive glass, *CBVA* Chitosan/Bioactive glass/vancomycin, *CBME* Chitosan/Bioactive glass/Melittin, *CBVME* Chitosan/Bioactive glass/vancomycin/Melittin

### Surface characterization

Scanning electron microscopy (JSM-7610F, JEOL Co., Japan and MIRA3, TESCAN Co., Czech) was employed for visualization of coatings’ morphology and microstructure. The surface wettability was assessed via static contact angle estimations by a DSA30 device (Kruess GmbH, Germany). Every sample was measured in triplicate by the use of Milli-Q water.

The interactions between the coatings constituents were determined by Fourier transform infrared spectroscopy (FTIR, ABB BomemMB100, USA) ranging from 400 to 4000 cm^−1^ with a resolution of 4 cm^−1^.

### Cell culture assays

#### Cell culture

Preosteoblast MC3T3-E1 cell line was procured from the national cell bank (Pasteur Institute of Iran, Tehran, Iran) and grown in α-MEM enriched with 10% fetal bovine serum, 100 IU/ml of penicillin, and 100 µg/ml of streptomycin at 37 °C in a moistened ambience with 5% CO_2_ for proliferation. To investigate differentiation, 1% L-ascorbic acid-2-phosphate 20 mM, 1% β- glycerophosphate 1 M and 0.00004% dexamethasone (1 mg/ml) were added to the proliferation medium. The confluent cells were taken up through trypsinization of attached cells and suspended anew in medium. Cells were counted by the trypan blue assay.

#### Cell proliferation test

Cell proliferation was assessed with the standard alamar Blue test procedure. In brief, 10,000 cells were seeded on the samples followed by incubation at 37 °C in 5% CO_2_ within 1, 3, and 7 days. Following each time point, the medium in wells was discarded, each well was supplied with fresh medium comprising 10% of 440 mM sterile resazurin solution in PBS, and the cells underwent incubation for 4 h. Absorbance was measured for each specimen at 570 nm with a reference wavelength of 630 nm by an ELISA reader (BioTek microplate reader, USA).

#### Alkaline phosphatase activity and DNA content tests

The samples in differentiation medium received MC3T3 cell seeding, which was replaced by fresh medium every 2 days after cell culture. Alkaline phosphatase (ALP) activity was measured on days 3, 7, and 14 after cell culture over samples. In each interval, cells were exposed to 0.5% Triton X-100 treatment in PBS and then frozen at −80 °C for three freeze-thaw cycles. The solution of samples underwent ALP activity assay by an ALP assay kit (pNPP: Sigma-Aldrich). Enzymatic activity was standardized against the total DNA content measured with Picogreen kit (Invitrogen) according to the company’s protocol.

### Antibacterial assay

#### Determination of antimicrobial potential of the experimental groups

Antimicrobial activity of the samples was examined against both MRSA and VRSA bacterial strains isolated earlier from burn wound of two patients hospitalized at a university hospital. The 1.5 × 10^4^ CFU/ml bacterial suspension was seeded on the samples (three samples per groups) and placed in an incubator at 37 °C. The bacterial suspensions were incubated with MRSA and VRSA for 6 h followed by plating the residual planktonic bacteria on Mueller Hinton Agar (MHA) plate and the re-incubating nightlong at 37 °C. The numbers of resultant colonies were counted to assess bacterial vitality. For attached bacteria, the bacterial suspension above samples were disposed of and substituted by 200 µL of PBS. The samples experienced sonication for 30 s in PBS for suspension of the attached bacteria from surfaces and the suspension was cultured on MHA as mentioned above.

#### Morphological assessment of bacteria

The bacterial cell morphology alterations resulting from the effects of samples on a VRSA strain were visualized using SEM method. Bacterial cell were fixed by 2.5% glutaraldehyde in PBS and then desiccated by 10, 30, 50, 70, 90, and 100% ethanol solutions, respectively. The samples were coated with gold nanoparticles and made visible using a FE-SEM device.

### Statistical analysis

Data are represented as mean ± standard deviation (SD) and results were subjected to statistical analysis with ANOVA using Tukey’s post-hoc *t*-test.

## Results and discussion

### Characterization of surfaces

**Fig. 1:**
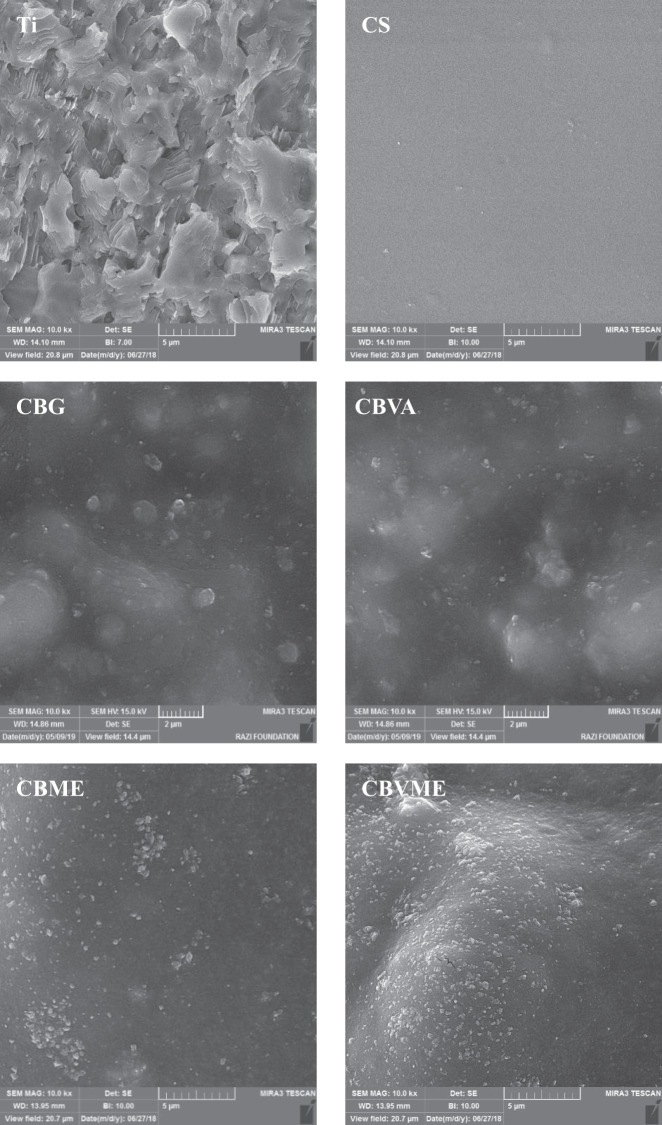
FE-SEM images of Ti: Titanium, CS: Chitosan, CBG: Chitosan/Bioactive glass, CBVA: Chitosan/Bioactive glass/vancomycin, CBME: Chitosan/Bioactive glass/Melittin, CBVME: Chitosan/Bioactive glass/vancomycin/Melittin coatings

**Fig. 2:**
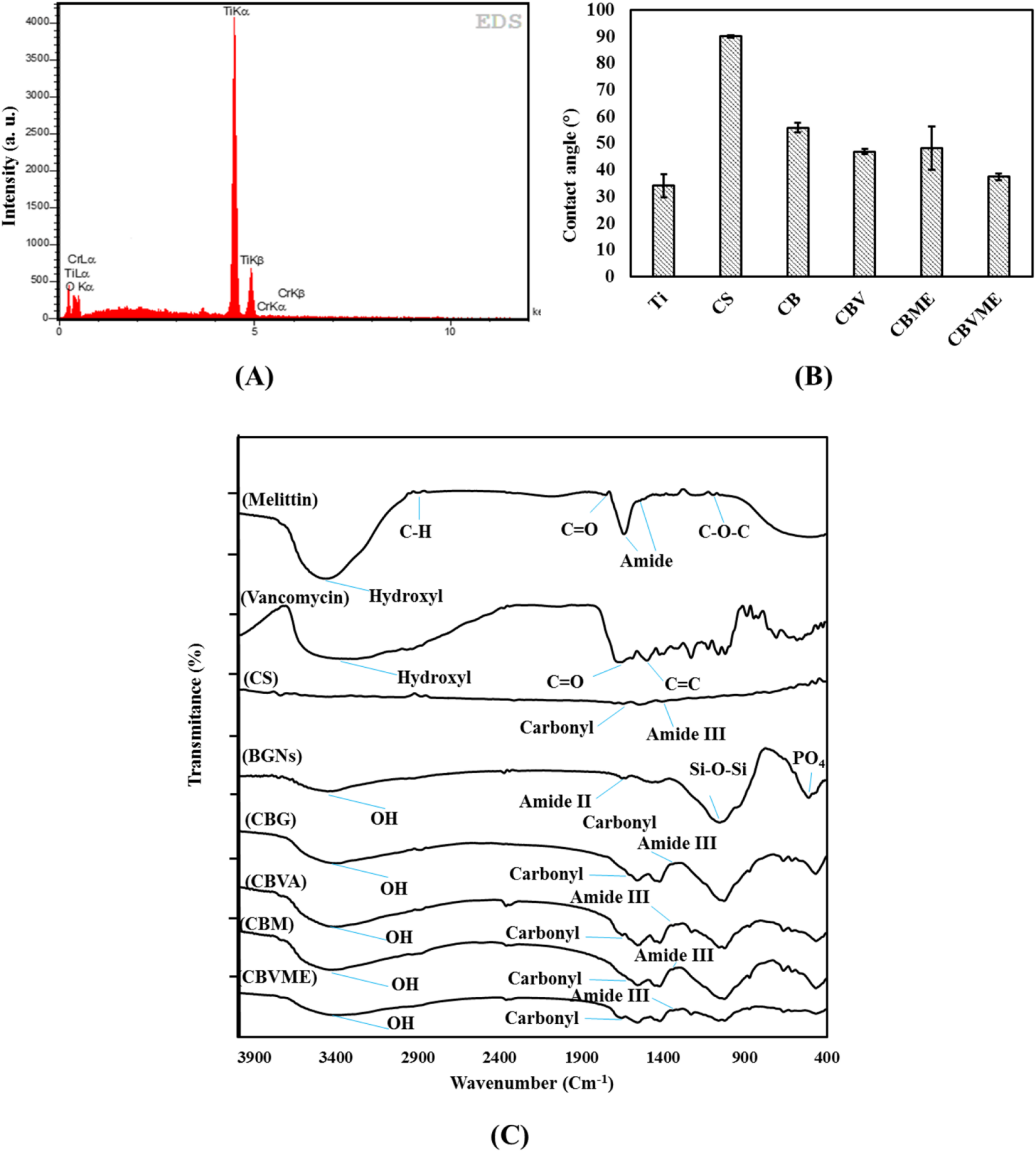
**A** EDS spectrum of Ti specimen after hydrothermally etching; **B** Contact angle of experimental groups; **C** FTIR spectra of pristine components and composite coatings (Ti: Titanium, CS: Chitosan, CBG: Chitosan/Bioactive glass, CBVA: Chitosan/Bioactive glass/vancomycin, CBME: Chitosan/Bioactive glass/Melittin, CBVME: Chitosan/Bioactive glass/vancomycin/Melittin)

FE-SEM images of hydrothermal-etched titanium substrate and the morphology of composite coatings are shown in Fig. 1. Two-dimensional constructs (plate-like) were noticed throughout the Ti samples treated hydrothermally. CS coating is smooth and uniform morphologically. The morphologies of CB, CBVA, CBME, and CBVME deposits reflected that BGN nanoparticles were dispersed in a polymer matrix. Figure 2A depicts the chemical composition of the surface resulted from the EDS spectra. Surface analysis revealed that only Ti and O elements were noticeable, suggesting that TiO_2_ phase were formed on Ti specimen surface. The water contact angle of the experimental groups is offered in Fig. 2B. A nearly 34° contact angle of etched Ti surface is fewer than those of chitosan and chitosan composite coatings. The contact angle of CB composite coating was statistically lower than CS coatings. The contact angle in CBVA, CBME, and CBVME coatings was lowered by vancomycin and Melittin in coatings.

The FTIR spectra of CS, CB, CBVA, CBME, and CBVME coatings and their components are presented in Fig. 2C. The functional groups of vancomycin, bioactive glass, and chitosan were reflected in composite coatings but Melittin functional groups were not found in composite coatings. As there was minor Melittin content, its functional groups did not appear in FTIR spectra of CBME and CBVME coatings. The wide bonds at 3450, 2900, 1659, and 1320 cm^−1^ are attributable to hydroxyl group, C-H stretching, carbonyl group, and amide III bond, respectively [16, 17]. The absorption bonds at 1055 and 877 cm^−1^ are related to Si-O-Si group in composite coatings [18]. The absorption bond at about 467 cm^−1^ corresponds to PO_4_ [18]. The organic functional groups of vancomycin were situated at 3340 cm^−1^ (phenolic OH), 1659 cm^−1^ (C=O stretching), and 1492 cm^−1^ (aromatic C=C stretching) [18]. Slight variations were observed in the peak locations of bioactive glass, chitosan, and vancomycin in CB, CBVA, CBME, and CBVME composite coatings. These are attributable to establishment of hydrogen bonds between hydroxyl and amine groups of CS and vancomycin and oxygen of the glass network [19].

These composite coatings displayed uniform morphology with no cracks or pores, leading to the conclusion that rather uniform coatings were formed as a result of the sedimentation procedure. Evaporation of water from acidic solutions of chitosan results in easy formation of robust chitosan/bioactive glass nanoparticles composite uniform coatings. This is because hydrogen bonds are present between the hydroxyl and amino groups of chitosan and TiO_2_ layer on the etched surface and oxygen of the bioactive glass network [20].

### Proliferation and differentiation of MC3T3-E1 cells by coatings

**Fig. 3:**
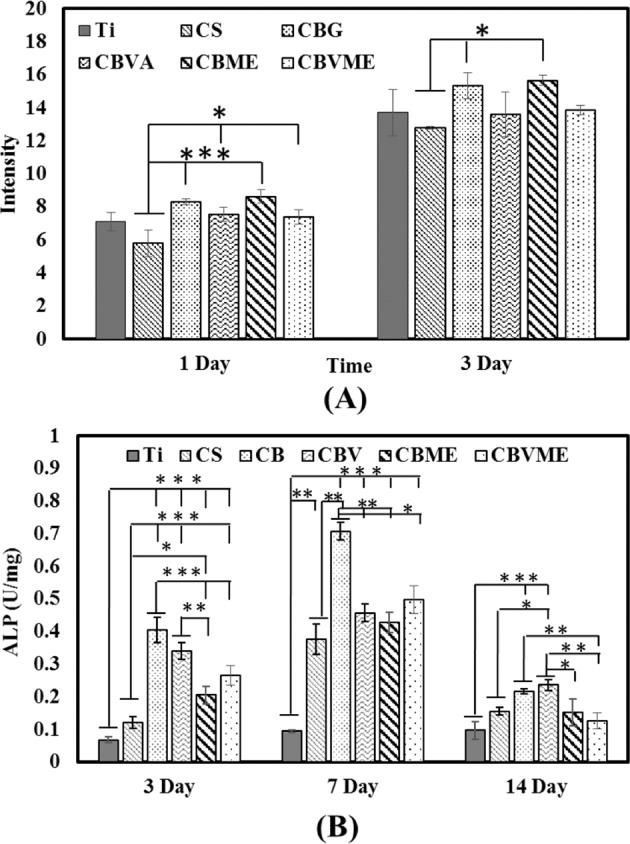
**A** Alamar Blue assay of experimental groups after 1 and 3 days. **B** Normalized ALP assays of MC3T3 cells cultured on surfaces after 3, 7 and 14 days. (*: *P* < 0.05) (Ti: Titanium, CS: Chitosan, CBG: Chitosan/Bioactive glass, CBVA: Chitosan/Bioactive glass/vancomycin, CBME: Chitosan/Bioactive glass/Melittin, CBVME: Chitosan/Bioactive glass/vancomycin/Melittin)

The proliferation of MC3T3 cells on experimental groups was examined by Alamare Blue test. As shown in Fig. 3A, cell proliferation for coatings was not significantly different in comparison to the substrate after 1 and 3 days of incubation. But, cells grown on CBG and CBME composite coatings underwent more rapid than those grown on CS group. The cell proliferated less in the CS group than Ti group, though, the two groups were not significantly different (Fig. 3A). Moreover, bioactive glass in composite coatings rendered acceleration of cell proliferation in composite coatings. Melittin in composite coatings affected positively cell proliferation that may be because of its high surface activity. It is mainly hydrophobic section in the terminal region of the molecule with the Lys-Arg-Lys-Arg sequence [4].

According to Fig. 3B, MC3T3 cells cultured on composite coatings had a significant secretion of ALP compared to other groups after 3 days. All coatings presented more ALP secretion than Ti group after 7 days. Throughout 14 days, all groups had less ALP concentrations (Fig. 3B). The present findings reliably indicate that composite coating caused differentiation of MC3T3 to osteoblasts, which slightly impaired following addition of vancomycin and Melittin as coating ingredients.

Bioactive glass nanoparticles were utilized in here as osteogenic materials. Bioactive glasses bond to bone and induce new bone growth with confirmed osteogenic function. Osteogenic features of bioactive glass improve with reduction of bioactive glass particle size, thereby, it raised ALP enzyme of MC3T3 cells and led to their differentiation to osteoblast cells. Melittin-mediated rise of proliferation in MC3T3 cells demonstrates that it is an appropriate option as osteoinductive and antibacterial agent in coatings of orthopedic implants. The proliferation and ALP enzyme of MC3T3 cells slightly decreased as a result of vancomycin application, so vancomycin had no positive effect on bone regeneration [21].

### Antibacterial performance

**Fig. 4:**
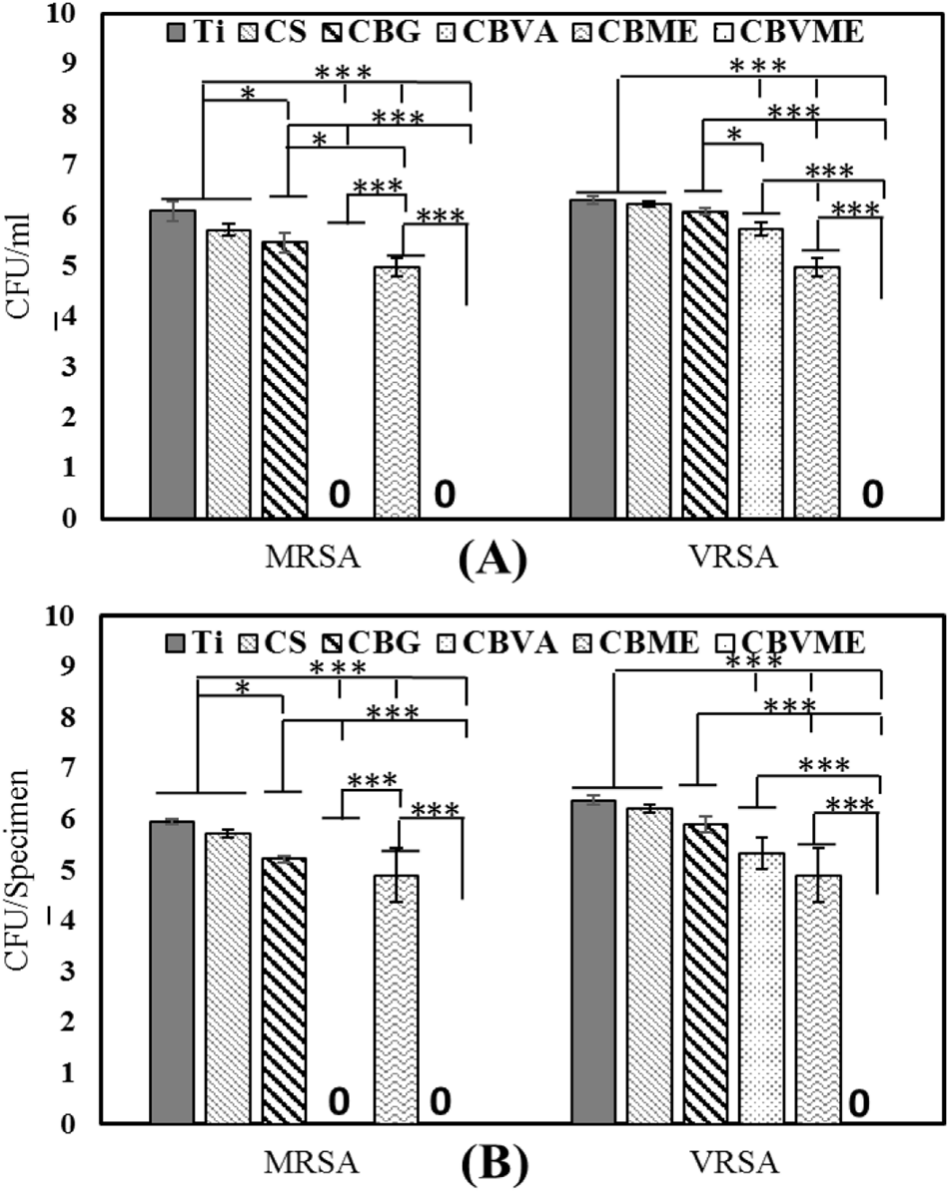
**A** Number of planktonic bacteria; **B** Number of adhered bacteria on different surfaces (*:*P* < 0.05, **:*P* < 0.01, ***:*P* < 0.001) (Ti: Titanium, CS: Chitosan, CBG: Chitosan/Bioactive glass, CBVA: Chitosan/Bioactive glass/vancomycin, CBME: Chitosan/Bioactive glass/Melittin, CBVME: Chitosan/Bioactive glass/vancomycin/Melittin)

**Fig. 5:**
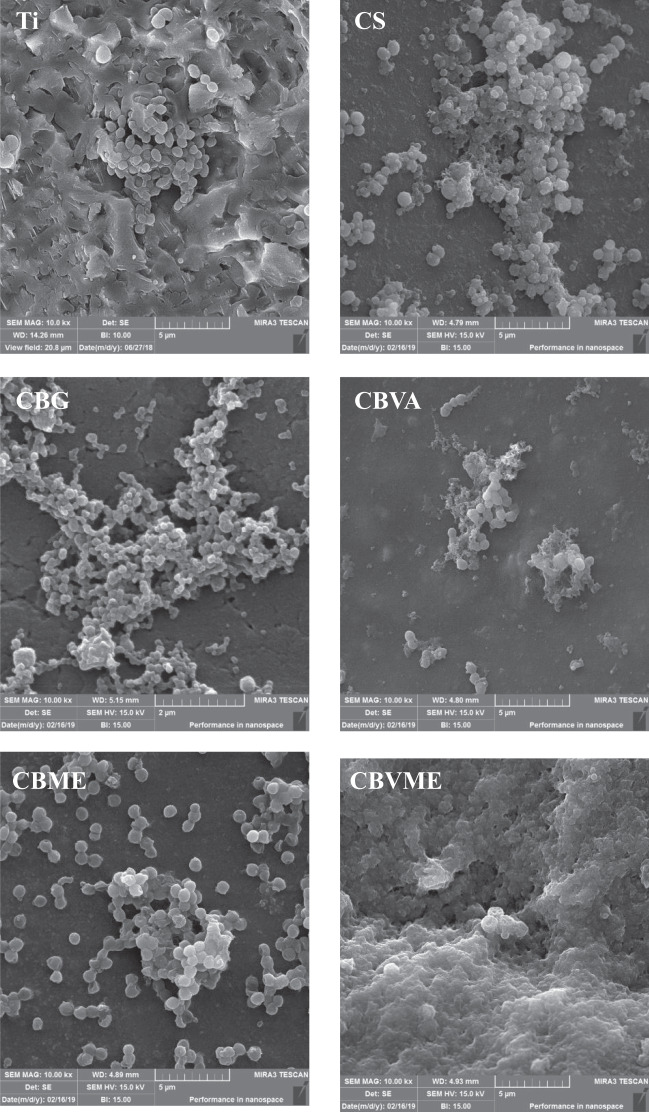
SEM images of VRSA bacteria on Ti: Titanium, CS: Chitosan, CBG: Chitosan/Bioactive glass, CBVA: Chitosan/Bioactive glass/vancomycin, CBME: Chitosan/Bioactive glass/Melittin, CBVME: Chitosan/Bioactive glass/vancomycin/Melittin coatings after incubation for 6 h

Both planktonic and adherent MRSA and VRSA bacteria were examined for appraising antibacterial activities of the experimental groups after 6 h (Fig. 4A, B). CBVME coating could eradicate both planktonic and adherent MRSA and VRSA bacteria due to the synergetic impact of vancomycin and Melittin. CBVA coating killed MRSA bacteria but it failed to abolish VRSA bacteria. Reductions were observed in both planktonic and attached bacteria in CS, CBVA, and CBME coatings versus Ti group. Figure 5 displays SEM images of VRSA bacteria on the surfaces within 6 h. Demolished bacteria are visible on CBVA, CBME and CBVME coatings along with live bacteria. Ti, CS, and CB coatings demonstrated morphologically normal live VRSA bacteria with no eradication of bacteria.

The “race for the surface,” phrase denotes tissue cell integration and bacterial attachment competition for settlement on the implant’s surface. It is necessary for implants to have anti-adhesive or bactericidal surfaces in early stages. The synergistic role of Melittin and vancomycin formed anti-adhesive or bactericidal surfaces that could kill both planktonic and adherent bacteria. Thus, biofilm development was hindered in initial phases along with occurring settlement of bone tissue cells. To kill bacteria, Melittin destroys bacterial cell membrane whereas vancomycin eliminates bacteria by interrupting the biosynthesis of bacterial cell-wall [22, 23]. Accordingly, it can be concluded that cell membrane is destroyed by Melittin leading to interruption of bacterial cell-wall biosynthesis by vancomycin. Chitosan and bioactive glass possess antibacterial activities influencing bactericidal features of coatings [19]. Amino groups of chitosan interrupt the bacterial membrane and eliminate them [24]. Alkaline ions releasing from bioactive glass raise the pH of medium, which is not tolerable by bacteria resulting in their eradication [25].

## Conclusions

In the present study, chitosan/bioactive glass nanoparticles/vancomycin composite coatings casted on hydrothermally etched titanium substrate. Then, the samples were covered by Melittin through drop casting technique. The addition of vancomycin or Melittin in the coatings led to significant reductions of both planktonic and adherent MRSA and VRSA bacteria. Combination of vancomycin and Melittin in the coatings eliminated both planktonic and adherent MRSA and VRSA bacteria very fast and inhibited biofilm development on the implant’s surface. Proliferation of MC3T3 cells was on the rise by the inclusion of Melittin in chitosan/bioactive glass composite coating. The inclusion of vancomycin could not positively affect ALP enzyme secretion.
